# Supplementary material for: Inputs for optimizing selection platform for milk production traits of dairy Sahiwal cattle
Source: PLoS One. 2022 May 23;17(5):e0267800. doi: 10.1371/journal.pone.0267800 (PMC9126386; doi:10.1371/journal.pone.0267800)
Supplement: S1 File — (PDF) [file pone.0267800.s001.pdf]

DATAFILE

sahiwal_data.txt

TRAITS

10

FIELDS_PASSED TO OUTPUT

1

WEIGHT(S)

RESIDUAL_VARIANCE

0.70

EFFECT

4 cross alpha

EFFECT

5 cross alpha

EFFECT

6 cross alpha

EFFECT

1 cross alpha

RANDOM

animal

OPTIONAL

pe

FILE

Ped.txt

FILE_POS

1 2 3 0 0

PED_DEPTH

0

(CO)VARIANCES

0.25

(CO)VARIANCES_PE

```
0.15
OPTION missing -999
# BLUP options
OPTION conv_crit 1d-12
OPTION maxrounds 10000
OPTION EM-REML 10
OPTION sol se
OPTION residual
OPTION solv_method FSPAK
OPTION use_yams
OPTION tol 1d-20
# Single Variances
OPTION se_covar_function a_1 G_4_4_1_1
OPTION se_covar_function pe_1 G_5_5_1_1
OPTION se_covar_function e_1 R_1_1
# Total Variance (sum genetic, permanent environmental, and residual variances)
OPTION se_covar_function P_1 G_4_4_1_1+G_5_5_1_1+R_1_1
# Ratios (heritability additive over total)
OPTION se_covar_function H2 G_4_4_1_1/(G_4_4_1_1+G_5_5_1_1+R_1_1)
OPTION se_covar_function R2 (G_4_4_1_1+G_5_5_1_1)/(G_4_4_1_1+G_5_5_1_1+R_1_1)
```
