# Supplementary material for: Inputs for optimizing selection platform for milk production traits of dairy Sahiwal cattle
Source: PLoS One. 2022 May 23;17(5):e0267800. doi: 10.1371/journal.pone.0267800 (PMC9126386; doi:10.1371/journal.pone.0267800)
Supplement: S2 File — (PDF) [file pone.0267800.s002.pdf]

```
DATAFILE
production_data.prn
TRAITS
4
FIELDS_PASSED TO OUTPUT
1
WEIGHT(S)
RESIDUAL_VARIANCE
0.7
EFFECT
2 cross alpha
EFFECT
3 cross alpha
EFFECT
1 cross alpha
RANDOM
animal
FILE
Ped.prn
FILE_POS
1 2 3 0 0
PED_DEPTH
0
(CO)VARIANCES
0.3
# BLUP options
OPTION missing -999
OPTION conv_crit 1d-12
OPTION maxrounds 10000
OPTION EM-REML 10
OPTION sol se
```

```
OPTION residual
OPTION solv_method FSPAK
OPTION use_yams
OPTION tol 1d-20
# Single Variances
OPTION se_covar_function a_1 G_3_3_1_1
OPTION se_covar_function e_1 R_1_1
# Total Variance (sum genetic and residual variances)
OPTION se_covar_function P_1 G_3_3_1_1+R_1_1
# Ratios (heritability additive over total)
OPTION se_covar_function H2 G_3_3_1_1/(G_3_3_1_1+R_1_1)
```
