# Supplementary figures and images for: Inputs for optimizing selection platform for milk production traits of dairy Sahiwal cattle
Source: PLoS One. 2022 May 23;17(5):e0267800. doi: 10.1371/journal.pone.0267800 (PMC9126386; doi:10.1371/journal.pone.0267800)

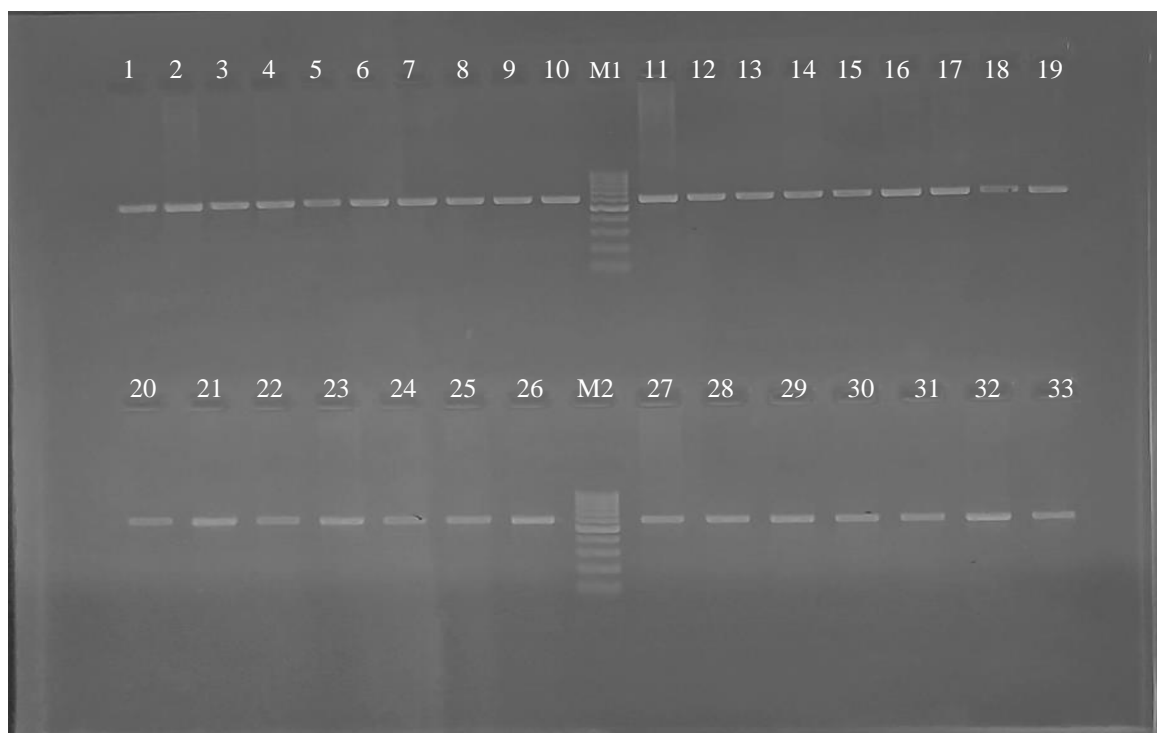

Supplement: S1 Raw image — The original gel image data was captured by a UV trans illuminator gel documentation system and saved as tiff file. Lane 1–33: PCR product (583 bp). Lane M1, M2: 100 bp DNA marker. (PDF) [file pone.0267800.s004.pdf]

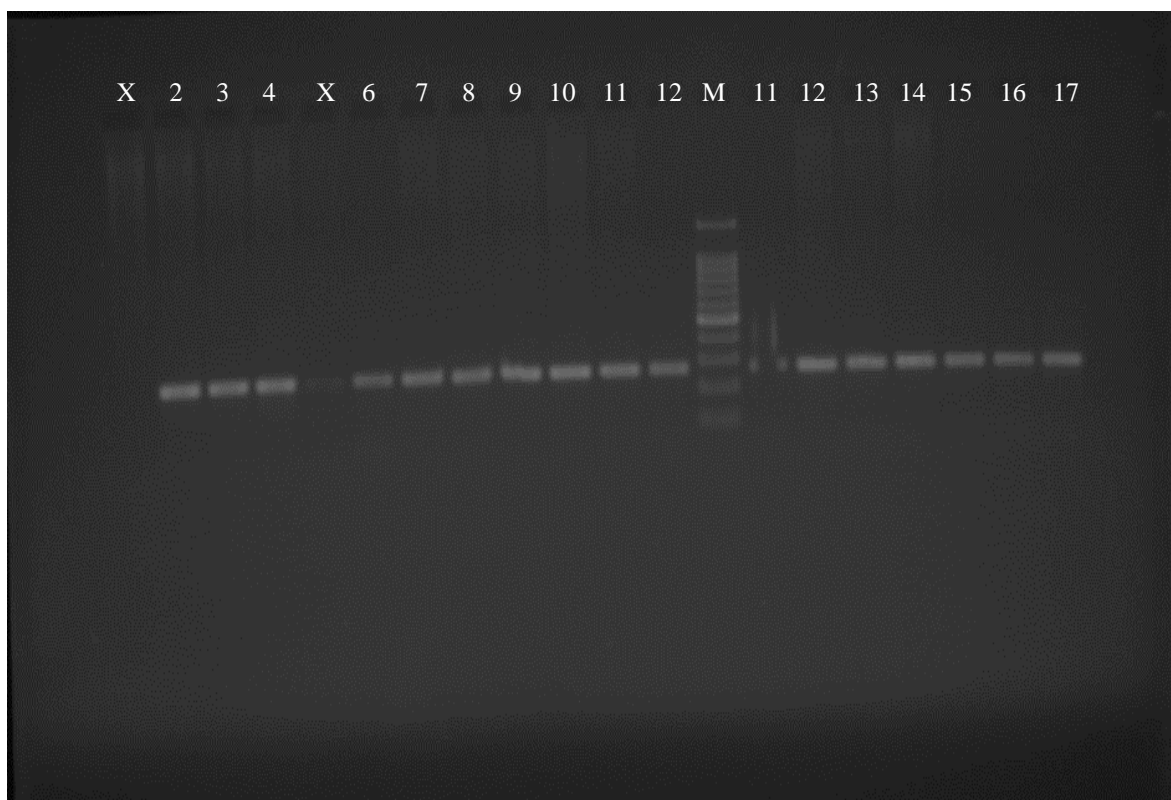

Supplement: S2 Raw image — The original gel image data was captured by gel documentation system (Gel.LUMINAX) and saved as tiff file. The lanes marked with “X” are non-specific amplifications and these experimental samples are not included in further genotyping purpose. Lane 2–17: PCR product (273 bp). Lane M: 100 bp DNA marker. (PDF) [file pone.0267800.s005.pdf]

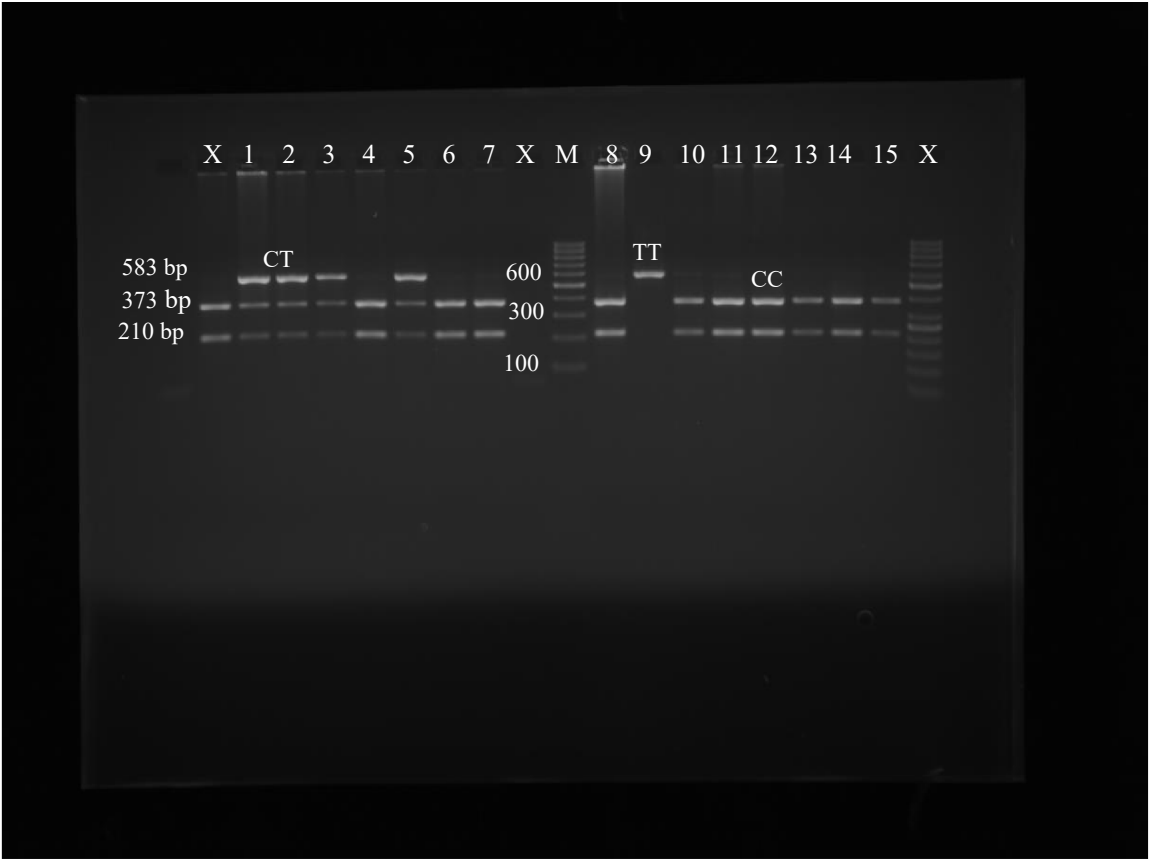

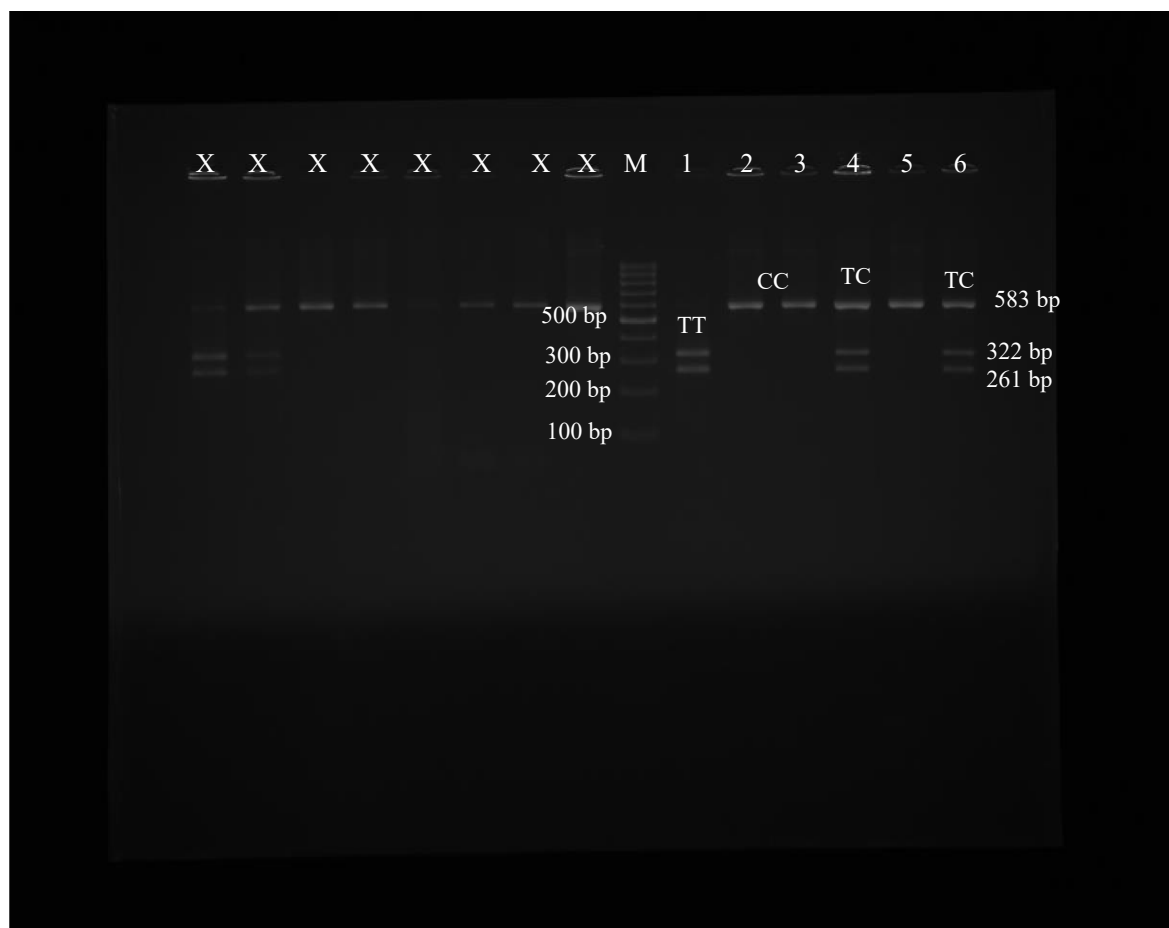

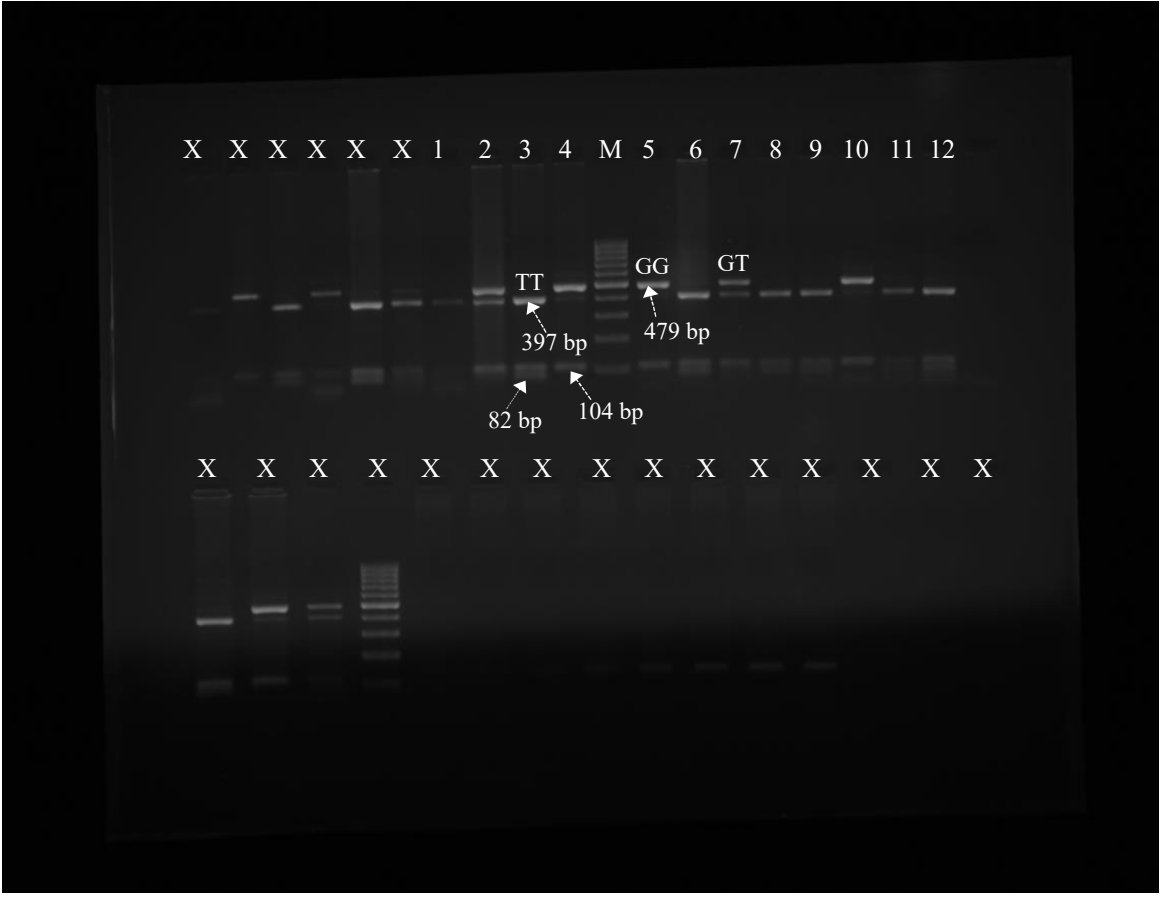

Supplement: S3 Raw image — (A) The PCR products of MASP2 gene at the SNP g.499C>T locus in Fig 1C was digested with BstUI enzyme and the digested products were separated by 2.8% agarose gel electrophoresis stained with ethidium bromide. The original gel image data was captured by a gel documentation system (Gel.LUMINAX) and saved as tiff file. The fragment sizes of the identified genotypes are shown in the left (583, 373 and 210 bp). Three genotypes were detected in this experimental population, namely CC (lanes 4, 6, 7, 8, 10–15), CT (lanes 1, 2, 3, 5) and TT (lane 9). Lane M is the 100 bp marker. The lanes not included in the final Fig 1C marked with “X” above the lane label on the original gel image. The final figure panel generated from the original image is shown in Fig 1C (left). (B) The PCR products of MASP2 gene at the SNP g.609T>C locus in Fig 1C was digested with BsrDI enzyme and the digested products were separated by 2.8% agarose gel electrophoresis stained with ethidium bromide. The original gel image data was captured by a gel documentation system (Gel.LUMINAX) and saved as tiff file. The fragment sizes of the identified genotypes are shown in the right (583, 322 and 261 bp). Three genotypes were detected in this experimental population, namely TT (lane 1), TC (lanes 4, 6) and CC (lanes 2, 3, 5). Lane M is the 100 bp marker. The lanes not included in Fig 1C marked with “X” above the lane label on the original gel image. The final figure panel generated from the original image is shown in Fig 1C (middle). (C) The PCR products of MASP2 gene at the SNP g.684G>T locus in Fig 1C was digested with BsrI enzyme and the digested products were separated by 2.8% agarose gel electrophoresis stained with ethidium bromide. The original gel image data was captured by a gel documentation system (Gel.LUMINAX) and saved as tiff file. Three genotypes, GG (lane 5), GT (lanes 2, 4, 7, 10) and TT (lanes 1, 3, 6, 8, 9, 11, 12) were detected in this experimental population. Lane M is the 100 bp marker. [file pone.0267800.s006.pdf]

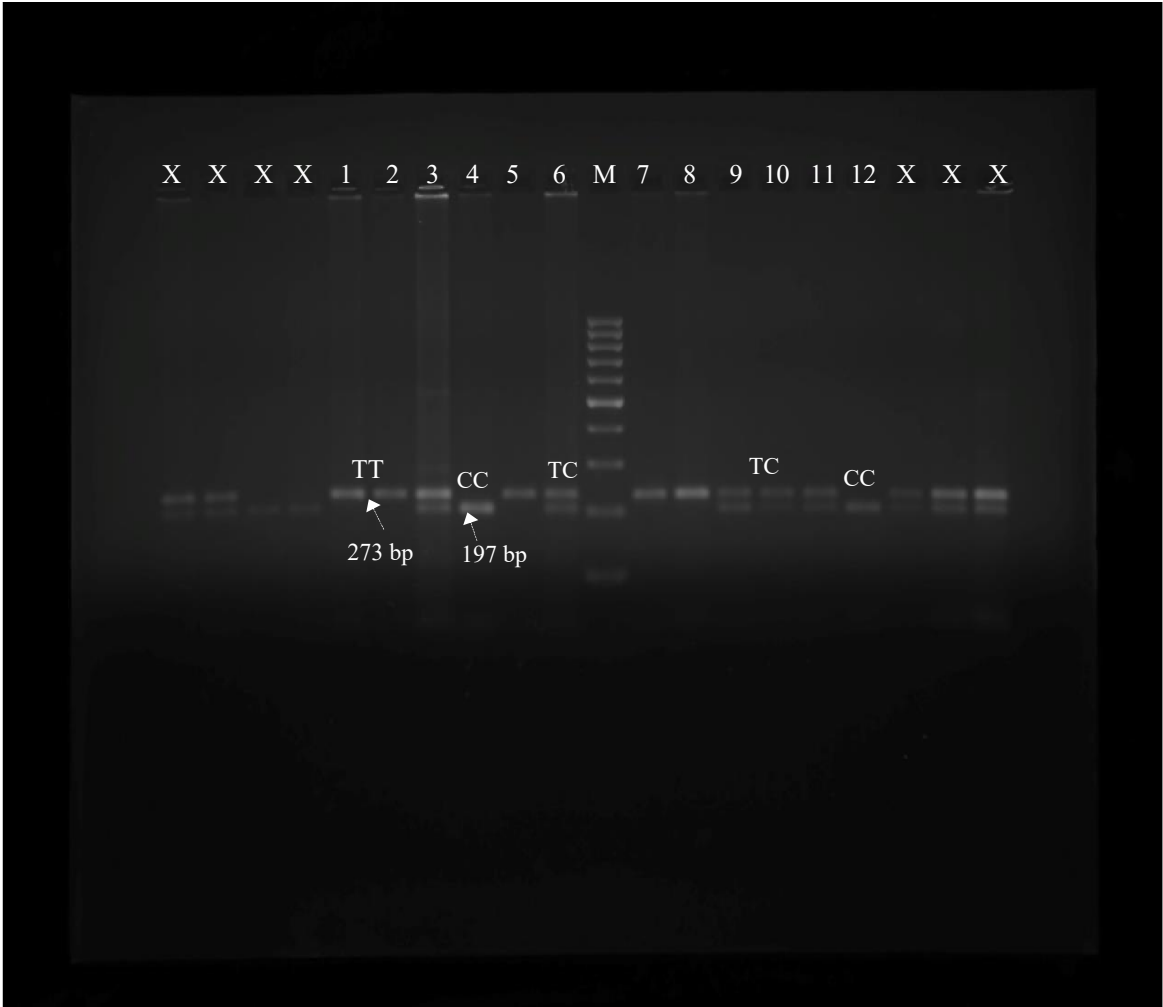

Supplement: S4 Raw image — The PCR products of the SIRT1 gene at the SNP g.-306T>C locus in Fig 2C was digested with BsaJI enzyme and the digested products were separated by 3% agarose gel electrophoresis stained with ethidium bromide. The original gel image data was captured by a gel documentation system (Gel.LUMINAX) and saved as tiff file. Three genotypes were detected in this experimental population, namely TT (lanes 1, 2, 5, 7, 8), TC (3, 6, 9–11) and CC (lanes 4, 12). For TC and CC genotypes, the fragment sizes of 39, 29 and 8 bp are invisible in the figure. Lane M is the 100 bp marker. The lanes not included in the final Fig 2C marked with “X” above the lane label on the original gel image. The final figure panel generated from the original image is shown in Fig 2C. (PDF) [file pone.0267800.s007.pdf]
