# Supplementary material for: Inputs for optimizing selection platform for milk production traits of dairy Sahiwal cattle
Source: PLoS One. 2022 May 23;17(5):e0267800. doi: 10.1371/journal.pone.0267800 (PMC9126386; doi:10.1371/journal.pone.0267800)
Supplement: Attachment — Submitted filename: review_statement.docx [file pone.0267800.s008.docx]

Abstract: Ok

This is a good piece of work. A comprehensive study on exploring the genetic architecture of milk production traits in dairy cattle is of great importance in livestock industry. However, if the following issues are addressed carefully, this work could have a great impact in improving productivity and save costs in dairy industry.

I suggest the author should undergo thorough revision (major) of the manuscript.

Introduction:

Regarding justification of the experiment, since the author mentioned (line number 101-103) very few studies have reported the genetic association of the SNP variation in MASP2 and SIRT1 gene with milk production traits. However, it would have been a novel work, if there was no previous report of association of these two genes with milk production traits.

Materials and method:

In line number 192, the author mentioned nine thousand and within the parenthesis mention 18000, which one is correct?

Results and discussion:

Line number 226: “in exon 3 region of” please replace ‘of’ with on.

Line number 233: “BstUI for MASP2 SNP at the locus” please change the line as “BstUI for MASP2 gene at the SNP locus”…………………

Line number 275: genetic diversity parameters and its corresponding Chi-squared test to determine….

Please mention the name of genetic diversity parameters such as expected heterozygosity, Observed heterozygosity, effective population size…

Line number 288-289: “At this locus the T allele frequency was minimal” which contradicts with that given in Table:3. In table 3, T allele is shown to have allele frequency of 0.81 for T allele and 0.19 for the allele C. The allele C’s frequency would be 0.81 and T allle’s frequency would be 0.19.

In line 299: The author described the genetic diversity in Shahiwal cattle as low PIC and low He indicating low genetic diversity. How will you explain the cause of low genetic diversity/ loss of genetic diversity in Sahiwal cattle? If the loss in diversity continues in this breed, this breed will face extinction. How would you solve this? Or what is your suggestion to improve the diversity? Please write in brief.

Line number 342: “Association analysis of candidate gene is a needful…..” Please rewrite or change like:

Association analysis of SNPs (variants) of candidate genes is a needful …………………….

Line 359-360: I find no reason to mention about the statistically non-significant SNPs in the text since it has been shown in the table. Please remove part of line 359-360…

Line 369: “In particular, TT genotype cows”………Please change to “In case of SIRT1 gene, TT genotype cows……

Line 370-371. It is suggested by the author to select heterozygotes (TC) for SNP, g-360T>C allele? How would you explain this higher performance of TC than CC and TT? Please write the explanation.

Line number 460-462: We know, usually, the repeatability value of a trait is higher or equal to the heritability values due to the differences in the calculation formula. In repeatability calculations we account for the permanent environmental effect. Therefore, higher repeatability value obtained in the present study might be attributed to much higher impact of permanent environment in the population. Please rewrite the sentences…………..

Line number 482, Please rewrite the sentence as : in general, EBVs for 305dmY, LMY and LL were increased………………

Line 485-486: The meagre change in response is due to low-selection intensity………………is in contrast to the line 280-282, where the author explained the reason for loss of genetic diversity due to increased selection pressure. Please rewrite the sentences and explain carefully.

Line 482: would you please explain the reason for very low genetic progress in LL, while all the milk production traits are highly correlated?

Line 482: The author did not focus on genetic trend for 305dSNFY and 305dFY in Sahiwal cattle. Please input the results somewhere in between. Please explain why didn’t author found any steady genetic trend for all the milk production traits.

Line 496-498: The figures are not found to be numbered in the figure section!!

The authors are claiming 3 SNPs found to be involved in the milk production of Shahiwal cattle. I would like to ask whether is it possible to calculate the portion of additive genetic variance captured by the mentioned SNPs? If possible, please include their additive genetic contribution as compared to the total variance. Since, the milk production traits are complex traits with lots of genes involved and they interact with the environment to express the phenotype, therefore, if the SNPs are not significantly contributing to the phenotype, the present study would have very little impact in improving milk production traits. Therefore, these days GWAS are being performed to identify genome-wide QTLs underlying milk production traits in livestock.

Conclusion:

Line 535 and 536, please rewrite to make the sentence more readable avoiding duplication in using “is of a …….”
